# Supplementary material for: Efficiency of the Cerebroplacental Ratio in Identifying High-Risk Late-Term Pregnancies
Source: Medicina (Kaunas). 2023 Sep 15;59(9):1670. doi: 10.3390/medicina59091670 (PMC10535994; doi:10.3390/medicina59091670)
Supplement: Supplementary file 1 [file medicina-59-01670-s001.zip › medicina-2573862-supplementary.pdf]

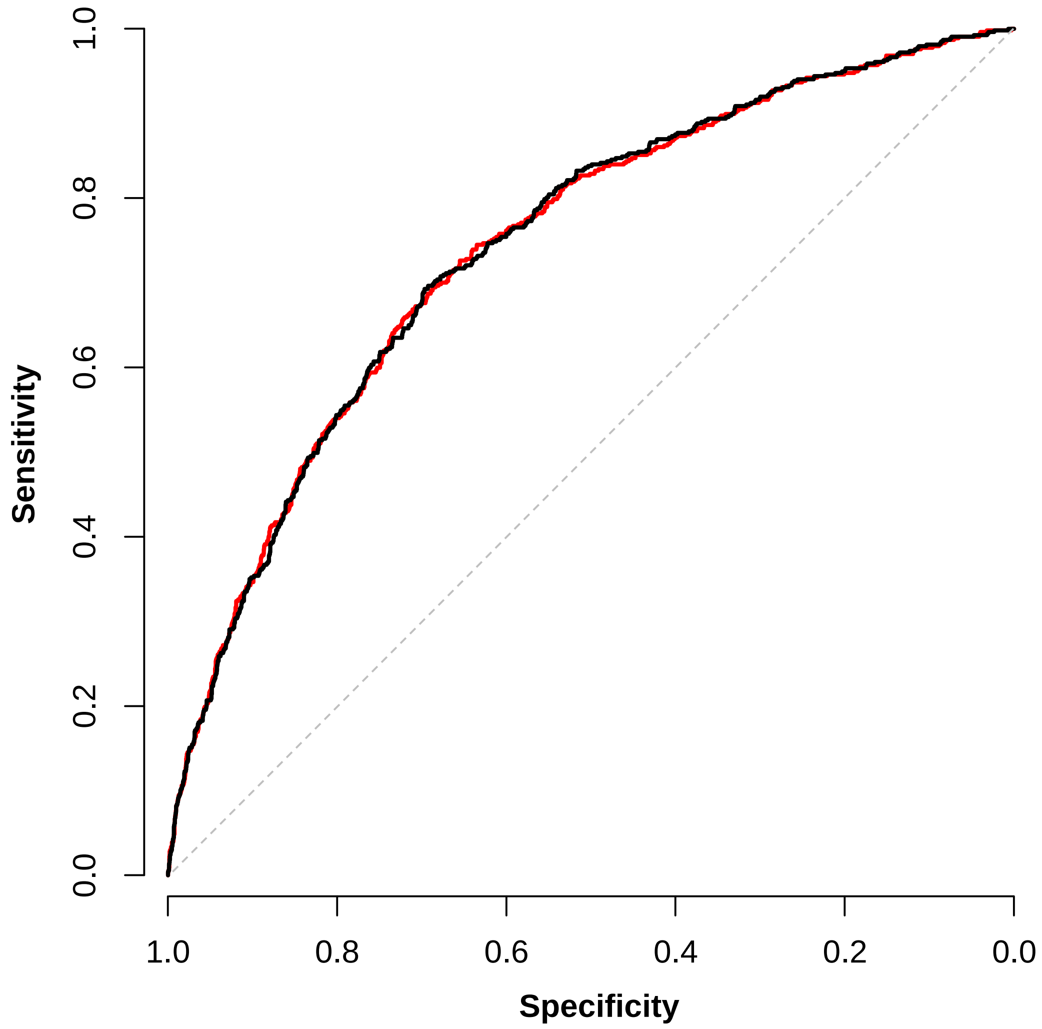

Figure S1. Receiver operating characteristics curves of the different models to predict adverse perinatal outcomes by maternal characteristics (red line) and maternal characteristics plus the cerebroplacental ratio (black line).
